# Systematic review of genome-wide gene expression studies of bipolar disorder

**DOI:** 10.1186/1471-244X-13-213

**Published:** 2013-08-15

**Authors:** Fayaz Seifuddin, Mehdi Pirooznia, Jennifer T Judy, Fernando S Goes, James B Potash, Peter P Zandi

**Affiliations:** 1 Department of Psychiatry and Behavioral Sciences, Johns Hopkins School of Medicine, Baltimore, MD, USA; 2 Department of Mental Health, Johns Hopkins Bloomberg School of Public Health, Baltimore, MD, USA; 3 Department of Psychiatry, University of Iowa Carver College of Medicine, Iowa City, IA, USA

**Keywords:** Microarray, Gene expression, Bipolar disorder, Mega-analysis, FKBP5, WFS1, Genome-wide, Brain, Prefrontal cortex, Hippocampus

## Abstract

**Background:**

Numerous genome-wide gene expression studies of bipolar disorder (BP) have been carried out. These studies are heterogeneous, underpowered and use overlapping samples. We conducted a systematic review of these studies to synthesize the current findings.

**Methods:**

We identified all genome-wide gene expression studies on BP in humans. We then carried out a quantitative mega-analysis of studies done with post-mortem brain tissue. We obtained raw data from each study and used standardized procedures to process and analyze the data. We then combined the data and conducted three separate mega-analyses on samples from 1) any region of the brain (9 studies); 2) the prefrontal cortex (PFC) (6 studies); and 3) the hippocampus (2 studies). To minimize heterogeneity across studies, we focused primarily on the most numerous, recent and comprehensive studies.

**Results:**

A total of 30 genome-wide gene expression studies of BP done with blood or brain tissue were identified. We included 10 studies with data on 211 microarrays on 57 unique BP cases and 229 microarrays on 60 unique controls in the quantitative mega-analysis. A total of 382 genes were identified as significantly differentially expressed by the three analyses. Eleven genes survived correction for multiple testing with a q-value < 0.05 in the PFC. Among these were *FKBP5* and *WFS1*, which have been previously implicated in mood disorders. Pathway analyses suggested a role for metallothionein proteins, MAP Kinase phosphotases, and neuropeptides.

**Conclusion:**

We provided an up-to-date summary of results from gene expression studies of the brain in BP. Our analyses focused on the highest quality data available and provided results by brain region so that similarities and differences can be examined relative to disease status. The results are available for closer inspection on-line at Metamoodics [http://metamoodics.igm.jhmi.edu/], where investigators can look up any genes of interest and view the current results in their genomic context and in relation to leading findings from other genomic experiments in bipolar disorder.

## Background

Bipolar disorder (BP) is a serious mental illness with considerable public health implications. It affects 1-2% of the general population [1], and costs the United States approximately $78.6 billion dollars annually in direct and indirect costs [2]. It is clear from family, twin and adoption studies that genetic factors play an important role in BP. Family studies show that compared to the general population the risk of disease is 5–10 times greater in first-degree relatives of a proband with bipolar disorder, and estimates of its heritability from twin studies range from 80-90% [3]. Yet, despite the overwhelming evidence, the genetic causes of BP remain largely unknown. This is likely due to the fact that the etiology of BP is complex and probably involves multiple independent and interacting genetic factors [4].

Microarray technology provides a powerful tool for studying the genetic contribution to complex disorders [5]. It allows for the measurement of gene expression levels genome-wide in a range of tissues and across disease conditions. A number of studies have used this technology to examine expression differences in BP versus unaffected controls with the goal of identifying genes or pathways of genes that are up or down regulated in the disorder [6,7]. These studies have typically used RNA samples from either peripheral blood or brain tissue [8]. The advantage of the former is that it is relatively easy to collect from participants. However, it may not be the relevant tissue for psychiatric disorders, that presumably have origins in the brain, and there may be constitutive differences in gene expression between blood and the brain. By contrast, the brain is the relevant tissue to study for BP. The disadvantage of brain tissue is that it can only be collected after the participant is deceased, which may limit the ability to collect sufficiently large samples. Additionally, because of the relative instability of RNA, post mortem factors (for e.g. brain tissue pH, coma, respiratory arrest, hypoxia, seizures, dehydration, multiple organ failure, and head injury) may confound the relationship between measured expression levels and disease status [9,10]. As a result, findings from studies using brain tissue have largely been inconsistent.

In order to synthesize the current findings to increase accuracy, we carried out a systematic review of existing gene expression studies of BP in humans. Motivated by the consideration that studies with brain would be the most informative for the etio-pathogenesis of BP, we conducted a quantitative mega-analysis of those studies carried out with this tissue. By combining data across studies to increase the sample size and using consistent procedures to process and analyze the data, we sought to summarize the findings from these studies and clarify their relevance for BP. The findings from this analysis are made available on Metamoodics (http://metamoodics.igm.jhmi.edu), a bioinformatics resource that synthesizes the results from genomic experiments in mood disorders and displays them within their genomic context.

## Methods

### Literature search and data collection

We identified genome-wide gene expression array studies in BP by conducting a broadly cast literature search of the PubMed database through November 6, 2012 with the following keyword algorithm: *(bipolar depression OR bipolar disorder OR mood disorder OR affective disorder OR major depression) and (gene expression OR microarray)*. A total of 1,387 articles were returned. These were manually reviewed by looking at their titles, abstracts, keywords, and full text as needed to identify those that reported on a genome-wide study in BP in humans. We further searched the references of these articles to identify any other articles that were potentially missed by the initial PubMed search. In addition to the literature search, we also queried public microarray repositories including Gene Expression Omnibus (GEO) (http://www.ncbi.nlm.nih.gov/geo/) [11] and Array Express (http://www.ebi.ac.uk/arrayexpress/) [12]. We also consulted with clinicians and researchers in the field to identify other unpublished data sources.

For inclusion in the review, the study had to be a case–control genome-wide gene expression array study in BP in humans. For the quantitative mega-analysis, we included only those gene expression array studies carried out with Affymetrix GeneChip Human Genome Arrays (http://www.affymetrix.com/estore/browse/products.jsp?productId=131453#1_1). The overwhelming majority of studies were carried out with this popular platform, and this allowed us to more efficiently standardize the preprocessing algorithm, the analyses, the significance thresholds and annotation builds across all studies [13].

We completed an evidence table with the following information extracted from each of the included studies: (i) principal investigator/corresponding author; (ii) disorders included; (iii) sources of samples; (iv) total number of samples assayed; (v) brain region/RNA source; (vi) microarray platform; and (vii) PubMed ID.

We sought to obtain the raw gene expression array data by scanning the literature to identify GEO accession identifiers (ID) or links for downloadable feature-level extraction output (FLEO) files such as CEL files. If the main text did not contain an accession ID or a link to any FLEO files, we searched existing repositories and the research group’s laboratory web pages. If unsuccessful, we wrote to the authors. If multiple publications used overlapping data, we identified the most comprehensive dataset available.

### Data processing

**Figure 1:**
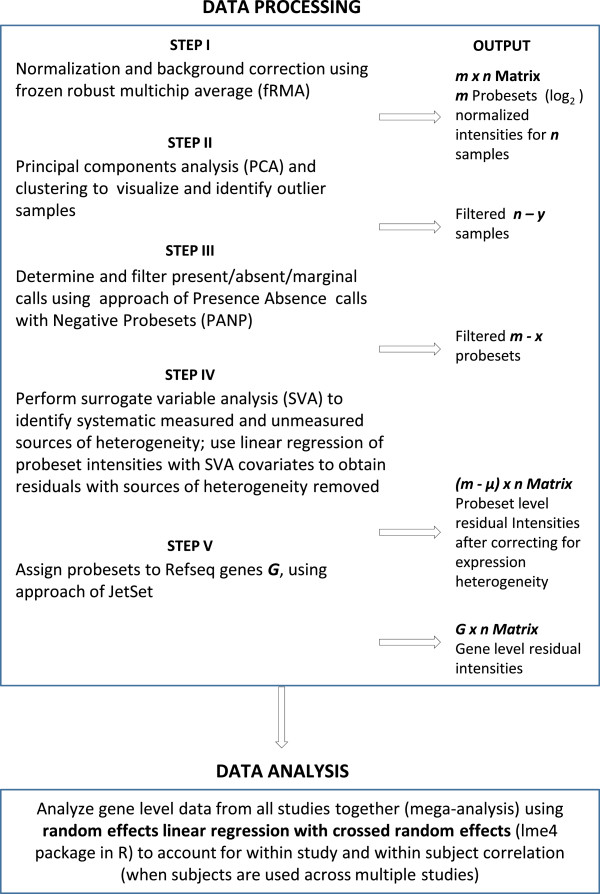
Workflow for data processing and analysis.

In order to consistently handle all datasets and eliminate bias introduced by relying on different algorithms used in the original studies, we obtained the raw data and converted these into analysis-ready gene expression data matrices (GEDM) by processing each study individually using a single analysis pipeline as illustrated in Figure 1. The processed GEDM’s for each study were then combined for the mega-analysis.

#### Step 1: Normalization and background correction using Frozen Robust Multi-array Analysis (fRMA)

CEL files obtained from each study (where each CEL file contained raw intensity values for thousands of probes/features from a single array hybridized to an individual sample) were pre-processed by applying normalization and background correction to the data. There are several established statistical methods for pre-processing raw gene expression array data. These can be categorized as multi-array, which require multiple samples/arrays to be analyzed simultaneously (e.g., MAS5 [14], RMA [15], gcRMA [16], MBEI [17], PLIER [18]), or single-array (e.g., fRMA [19]). Here, we used fRMA, which is a single-array preprocessing method that retains the `advantage of multi-array preprocessing. Briefly, fRMA uses publicly available gene expression array data on a specific array platform to create vectors or parameter estimates that are frozen. The basic idea is that the frozen parameter estimates are created from gene expression array datasets on diverse biological samples from a range of tissues and, therefore, capture the vast heterogeneity within and between samples/arrays. The frozen vectors are then used to pre-process (scale) a new gene expression array from the same platform. Because fRMA is specific to a single platform, a separate frozen vector must be created for each of the different platforms. For studies carried out with the Affymetrix U133A and U133 Plus 2.0 platforms, we used previously generated frozen vectors available as part of the frma package in R [20,21]. For studies carried out with the U95AV2 platform, we generated our own frozen vectors. We did this by downloading from GEO all gene expression array studies done on the U95AV2 platform using GEO Platform Accession: [GEO: GPL8300]. A total of 5,175 samples/arrays were returned on this platform. After filtering for *Homo sapiens* and querying the GEO database for the individual CEL files, a total of 2,633 samples/arrays were retained. These were grouped into experiments/batches based on their GEO Series ID. There were a total of 110 unique experiments/batches from diverse tissues ranging from 2–233 arrays/samples per experiment/batch. We retained all experiments/batches with greater than or equal to 5 samples/arrays, and used the frmaTools package [22] to build a frozen/fixed parameter vector with the makeVectorPackage.

After pre-processing each study using fRMA a matrix of normalized and background corrected (log_2_) intensities were obtained for each sample/array. These were aggregated for each study yielding an *m x n* study-specific matrix with *m* probesets and *n* samples/arrays.

#### Step 2: Removing outliers using Principal Component Analysis (PCA)

In this step, each *m x n* study matrix from the previous step was analyzed to identify and remove any poor quality samples/arrays. PCA and hierarchical clustering were used to visualize the relationship between samples/arrays and determine if any were outliers. The *boxplot, cor, sample covariance vs. sample means, prcomp,* and *hclust packages in R*[23] were used for sample/array quality control and visualization.

#### Step 3: Filtering probesets using Presence Absence Calls with Negative Probesets (PANP)

Here, individual probesets were filtered based on present/absent calls estimated using an algorithm denoted as PANP (Presence Absence Calls with Negative Probesets) [24]. Unlike present/absent calling algorithms such as MAS5 [14] which require both perfect match (PM) and mismatch (MM) probe data, the PANP algorithm was designed to analyze preprocessed data from PM probes only, such as was the case with our data. PANP takes advantage of so-called Negative Strand Matching Probesets (NSMPs), which are found on arrays when the Expressed Sequence Tag (EST) [25] data from which probeset sequences are created is conflicting, and probesets in both directions at the locus are included on the array. The NSMPs do not hybridize to any target, and thus provide a good proxy as a per-sample control for non-specific hybridization. Using Affymetrix annotation tables [26], we identified all probesets labeled as NSMPs that were not characterized with “cross hyb,” indicating the probeset may match to another gene. We then mapped the NSMPs to the ENSEMBL [27] transcript database version GRCh37. p8 and classified the NSMPs into four categories [28]: (i) probesets that did not map to a transcript at all; (ii) probesets that detected sense transcripts; (iii) probesets that detected antisense transcripts; and (iv) probesets that detected a sense transcript that overlaps with an antisense transcript.

For each gene expression *m x n* study matrix, we plotted the probability distribution of intensities using all probesets for visualization and quality control purposes. As expected, the NSMPs that did not map to a transcript at all were generally on the lower end of the intensity distribution. We used these confirmed NSMPs as input to the *panp* package in R [29]. Briefly, PANP uses the cumulative probability distribution of signal intensities calculated from the NSMPs relative to the remaining probesets to help define intensity cut-offs for calling a probeset as absent (A), marginal (M) or present (P). The thresholds for making these calls were selected to yield a false positive rate of 20% of calling a probeset as present when it is indeed absent. If a probeset was called as A for all subjects in an individual study, then that probeset was declared as absent from that study. We chose liberal cutoffs for filtering the probesets, because we wanted to maximize the number of probesets in each gene expression array study available for downstream analysis.

#### Step 4: Removing batch effects using Surrogate Variable Analysis (SVA)

SVA was carried out with each study to identify and remove systematic measured and unmeasured sources of variability other than case/control status, such as technical, genetic, environmental, or demographic factors [30-32]. These sources of heterogeneity are common in genome-wide gene expression studies, and failing to account for them in the analysis can obscure results. The SVA algorithm is performed in three steps: 1) the signal due to the primary variables of interest is removed and a residual expression matrix is obtained; 2) the subsets of genes driving signatures of expression heterogeneity remaining in the residuals are identified; and 3) surrogate variables for each subset of genes are generated.

We performed SVA on the *m x n* gene expression matrix from each study using the default iteratively re-weighted algorithm, and identified all significant surrogate variables. We then used these surrogate variables in a linear regression of each probeset intensity value and retained the residuals from this regression to generate a new *(m - μ) x n* matrix, where *(m - μ)* are the residual probeset intensities obtained for the *n* samples/arrays after removing the extraneous sources of heterogeneity. The final matrix of residuals was then used in the downstream steps.

#### Step 5: Mapping probesets to genes using JetSet

For each study, probesets were assigned to RefSeq genes using manufacturer annotation files confirmed as needed by mapping probe sequences to the human reference genome. The Affymetrix U133A, U133 Plus 2.0 and U95AV2 arrays have different design criteria that may lead to the creation of multiple probesets for the same gene [33]. To facilitate the synthesis of data across studies, we sought to assign the most representative probeset to each gene using a method implemented in JetSet [34]. JetSet considers three criteria for selecting the most representative probeset for each gene: 1) the specificity of probes in a probeset hybridizing to the target gene and not to other genes; 2) the extent to which the probeset covers different splice isoforms of the target gene; and 3) the distance of the probeset to the 3’ end of target transcripts as those that are closer to the 3’ end generally have stronger signal intensities due the initiation of transcription at the poly-A tail and are also more robust against transcript degradation. After resolving the mapping of maximally representative probesets to each gene, we ended up with a *G x n* matrix that contains *G* gene level residual intensities for *n* samples/arrays for each study.

### Data analysis

We combined the data for each study into a single large matrix and conducted a mega-analysis. We refer to this as a mega-analysis because the individual level data from each study were analyzed together instead of having been done separately by study and then having been summarized across studies as in a meta-analysis. Because of the challenges in obtaining brain samples, many studies used samples from the same brain collection. The mega-analysis approach allowed us to more efficiently address the overlap in samples by using mixed effects linear regression with crossed random effects for study and subject to account for both within study and within subject correlations [35]. We used the lmer function from the lme4 package [36] in R with default parameters to fit the mixed-effects models. The primary fixed effect of interested was a dichotomous variable for case–control status. Since the SVA was carried out to address measured and unmeasured confounding, we did not include other fixed effects covariates in the models. We fit separate models for each gene. Summary fold changes (FC) by case–control status, standard errors, 95% confidence intervals, p-values and false discovery rate q-values [37] for each gene were stored as output from each of the models. Volcano plots of the full results were graphed to visualize the significance of each gene with respect to pooled effect size/fold change. We identified significant differentially expressed genes as those with a regression beta estimate = ±0.1, which was equivalent to FC < −1.07 (down-regulated) or FC > 1.07 (up-regulated), and with p-values < 0.05. We used this relatively liberal threshold to maximize the inclusion of true differentially expressed genes in BP that may not always be among the most significant findings, at the risk of including some false positive associations.

Because gene expression studies in BP have been carried out with samples from several different brain regions, we conducted three separate mega-analyses of studies on: 1) any region of the brain; 2) the prefrontal cortex (PFC); and 3) the hippocampus. For the first two we included only studies done with the U133A and U133 Plus 2.0 platforms to minimize heterogeneity across studies and maximize the consistency of results using the most recent and comprehensive array data available, while for the hippocampus we included the one study done with the older U95AV2 array in order to have sufficient numbers for a combined analysis. We excluded one of the eligible studies carried out on the U133A platform because the results from it were widely and unaccountably divergent from the others as quantitatively shown in [38].

We used the program DAVID [39,40] to determine if there was an enrichment of common pathway annotations among the significant differentially expressed genes in the three mega-analyses (191 in any brain region, 160 in the PFC and 118 in the hippocampus). We used the default options and uploaded gene lists from each analysis separately as RefSeq gene symbols. Pathways included were the Biological Biochemical Image Database (BBID) [41], BIOCARTA and KEGG_PATHWAY [42]. Other annotation categories included were Gene Ontology [43] specifically GOTERM_BP_FAT which is the summarized version of biological processes in the Gene Ontology.

## Results

### Qualitative review

Additional file 1 lists all the genome-wide gene expression array studies on BP identified in our literature search. We found 30 genome-wide gene expression array case–control studies of BP [10,38,44-64]. Of these, only five examined just BP versus controls. The remaining 25 also included comparisons for cases with major depression, schizophrenia, and/or suicide. The 30 expression studies of BP examined tissue mainly from peripheral blood (n = 5) or brain (n = 25). The 25 studies of the brain used samples from a variety of regions including: the cerebellum (n = 3), frontal cortex (n = 15), orbitofrontal cortex (n = 1), primary visual cortex (n = 1), cingulate cortex (n = 1), parietal cortex (n = 1), anterior cingulate cortex (n = 2), locus coeruleus (n = 1), nucleus accumbens (n = 1), hippocampus (n = 4), and thalamus (n = 1). These numbers do not add to 25, because several studies examined tissue from multiple brain regions. The majority of these studies were done with samples obtained from one of four brain banks/resources: 1) the Stanley Medical Research Institute/Stanley Foundation (SMRI), which included samples from two different collections referred to as the Array Collection – SMRI (A) and Neuropathology Collection – SMRI (C) (data available at: https://www.stanleygenomics.org/); 2) the Harvard Brain Tissue Resource Center (McLean Hospital, Belmont, Massachusetts) (HBTRC) (data available at: http://national_databank.mclean.harvard.edu/brainbank/Main); 3) the Pritzker Neuropsychiatric Disorders Research Consortium (http://www.pritzkerneuropsych.org/); and 4) the Quebec Suicide Brain Bank (QSBB) (http://www.douglas.qc.ca/page/brain-bank). Raw expression data from the Pritzker Consortium and QSBB were not publically available and could not be obtained from the investigators. The overwhelming majority of the 25 studies on the brain were carried out with Affymetrix array platforms. Thirteen were carried out with the U133A or U133 Plus 2.0, seven on U95AV2, three on cDNA, one on Codelink, and one on Agilent arrays.

### Quantitative results

Table 1 lists the 10 genome-wide gene expression microarray studies that were included in the quantitative mega-analyses. We decided to focus on studies of the brain because this is arguably the most relevant tissue for a psychiatric disorder like BP. We carried out separate mega-analyses for three partially overlapping sets of studies done with samples from different regions of the brain in order to compare and contrast region-specific differences that may be relevant to disease. The three overlapping sets of studies included those on: 1) any brain region (n = 9); 2) the PFC (n = 6); and 3) the hippocampus (n = 2). The mega-analysis of any brain region included all studies on the PFC, one study on the hippocampus, and two additional studies on the anterior cingulate and thalamus. The mega-analyses of the PFC and the hippocampus were carried out with non-overlapping studies.

**Table 1:** Genome-wide gene expression studies of bipolar disorder with brain tissue samples included in the mega-analysis

**Study**	**Disorder**	**Source of samples**	**BP**	**CTRL**	**Brain region/RNA source**	**Genome-wide array platform**	**Total probesets**	**Probesets after P/A filter**	**After gene mapping**	**Included in mega-analysis**		
										**Any brain region**	**Prefrontal cortex**	**Hippocampus**
^**a**^**Altar A. et al.**	BP, SCZ	SMRI(A)	32	34	FrontalBA46	hgu133a	22283	15023	8962	YES	YES	-
**Bahn et al.**	BP	SMRI(A)	32	33	FrontalBA46	hgu133a	22283	15595	9214	YES	YES	-
^**b**^**T. Kato et al.**	BP, SCZ	SMRI(A)	33	34	FrontalBA46	hgu133a	22283	15972	9418	YES	YES	-
^**c**^**Dobrin et al.**	BP, SCZ	SMRI(A)	27	25	FrontalBA46	hgu133p	54675	40605	15995	YES	YES	-
**Laeng et al.**	BP, SCZ	SMRI(A)	20	21	Hippocampus CA1	hgu133p	54675	29854	13459	YES	-	YES
**Chen et al.**	BP, SCZ	SMRI(C)	14	13	FrontalBA46	hgu133p	54675	29854	12864	YES	YES	-
^**d**^**Kemether et al.**	BP, MD, SCZ	SMRI(C)	11	12	Thalamus MD	hgu133p	54675	30201	13464	YES	-	-
**Harvard_collection**	BP, SCZ	HBTRC	19	26	Frontal McL66	hgu133a	22283	15105	9021	YES	YES	-
**Harvard_collection**	BP, SCZ	HBTRC	15	21	Cingulate Cortex	hgu133a	22283	15264	8993	YES	-	-
**Harvard_collection**	BP, SCZ	HBTRC	8	10	Hippocampus	hgu95av2	12625	no filter	8438	-	-	YES

Number of outlier samples excluded from each study, ^a^ = 5, ^b^ = 2, ^c^ = 8, ^d^ = 1.

HBTRC – Harvard Brain Tissue Resource Center (McLean Hospital, Belmont, Massachusetts).

SMRI (A/C) - Stanley Medical Research Institute/Stanley Foundation (Array Collection/Neuropathology Collection).

Among the included studies, there were a total of 211 microarrays on 57 unique BP cases and 229 microarrays on 60 unique controls. On average, studies with the U133A and U133 Plus 2.0 arrays had data on 22,283 and 54,675 probesets, respectively, while those with U95AV2 had data on 12,625 probesets. After data processing there were on average 9,075 and 13,945 probesets for studies on U133A and U133 Plus 2.0, respectively, and 8,438 probesets for studies on U95AV2 that mapped to unique RefSeq genes.

**Figure 2:**
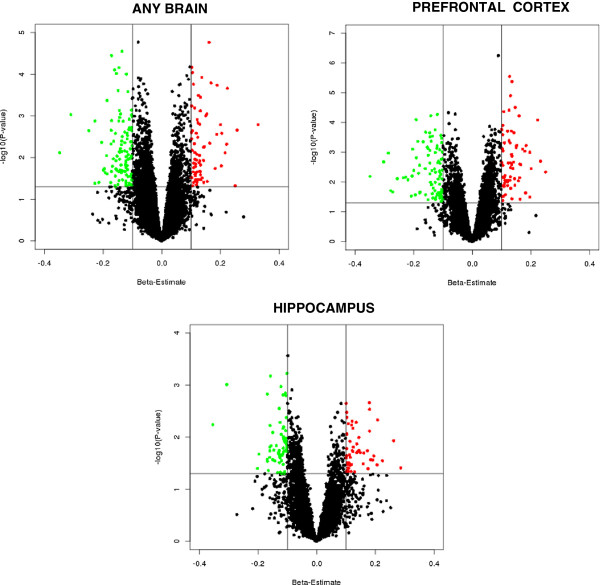
**Volcano plots showing effect size estimates by significance of each gene for the three mega-analyses.** Effect sizes captured as log_2_(FC) are shown on the X-axis, and significance levels measured as –log_10_(p-value) are shown on the Y-axis. Each dot represents an individual gene. Red dots represent significantly up-regulated genes with log_2_(FC) > 0.1 (FC > 1.07) at p-value < 0.05, while green dots represent significantly down-regulated genes with log_2_(FC) < -0.1 (FC < -1.07) at p-value < 0.05.

**Figure 3:**
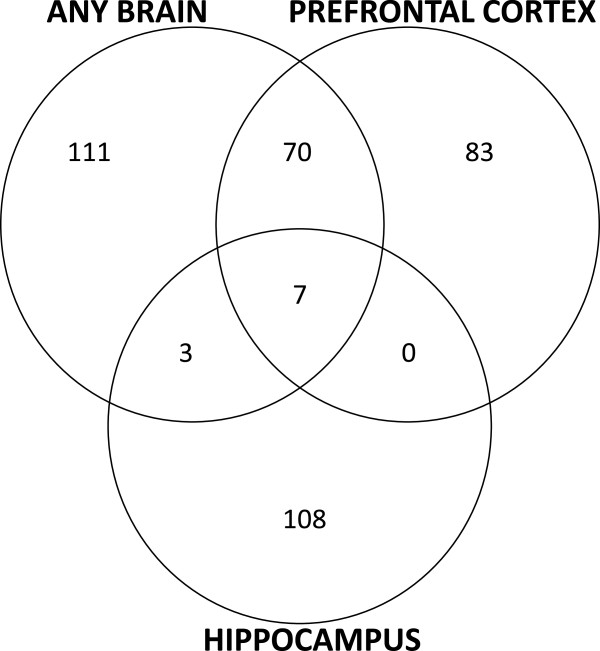
Venn diagram showing the concordance of 382 significant differentially expressed genes with a regression beta estimate = ±0.1, equivalent to fold change (FC) > 1.07 (up-regulated) or FC < −1.07 (down-regulated) with p-value < 0.05 from the three mega-analyses.

Figure 2 shows volcano plots for the results of the mega-analyses of studies on any brain region, the PFC, and the hippocampus. The red and green points represent the significant differentially expressed genes. The Venn diagram [65] in Figure 3 shows the overlap of the significant differentially expressed genes between the three mega-analyses. A total of 382 genes were identified: 191 in any brain region, 160 in the PFC, and 118 in the hippocampus; 80 of these were identified in more than one mega-analysis. Additional file 2 provides details of these 382 genes.

**Figure 4:**
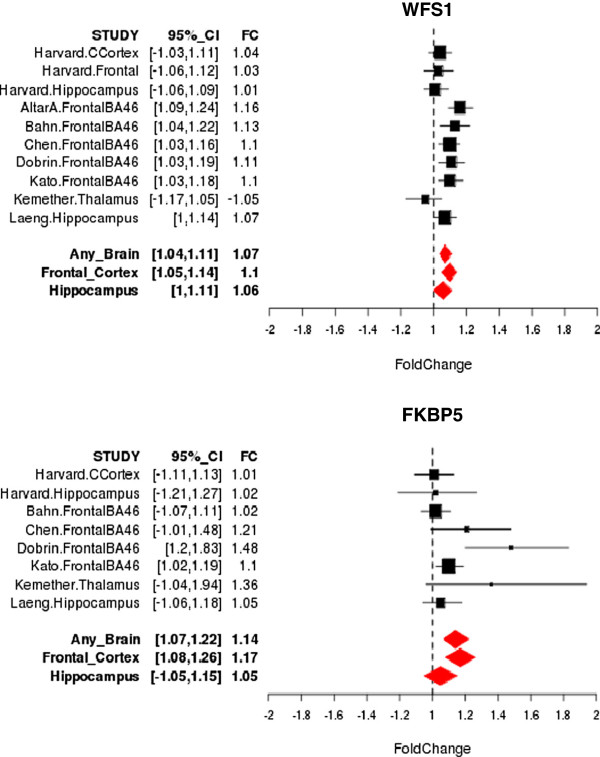
**Forest Plots of two genes of interest in mood disorders (q-value < 0.05) showing the estimated fold change (FC) of gene expression comparing BP cases and controls and 95% confidence interval for each study.** Summary estimates are provided for any brain regions, prefrontal cortex and hippocampus.

None of the genes identified as differentially expressed in any brain region or the hippocampus survived correction for multiple testing at a q-value threshold of 0.05. However, 11 genes had a q-value < 0.05 in the analysis of the PFC. Details of these 11 genes are highlighted in Table 2. Among these were two genes that have been previously implicated in mood disorders by numerous studies: *FKBP5* and *WSF1*. Figure 4 shows Forest plots for these two genes of interest. Although not as significant, there were a number of other notable candidate genes for mood disorders identified among the set of 382 differentially expressed genes. These included, for example: *DUSP6*, *CRH*, *NPY*, *NR4A2*, *SST*, *GRIK2*, *S100B* and *CACNA1C*. Several gene categories were identified with a Bonferroni corrected p-value < 0.05 across the three mega-analyses as shown in Additional file 3. For analyses of any brain and PFC regions, the most significant categories were related to metallothionein and metal-ion binding proteins. These findings were driven by a small collection of metallothionein genes, including predominantly *MT2A*, *MT1E*, *MT1H*, *MT1G*, and *MT1X*. Also among the top findings were MAP kinase phosphatase genes in the PFC, including the aforementioned DUSP6, and neuropeptide genes, including the aforementioned *NPY* and *SST,* in any brain, PFC and hippocampus. Interestingly, none of the metallothionein gene categories were identified in the analyses of the hippocampus samples.

**Table 2:** Summary results of 11 significant differentially expressed genes in bipolar disorder with a false discovery rate q-value < 0.05

**Genes**	**Fold change**	**95% CI**	**P-value**	**Q-value**	**Brain region**
**ALDH1L1**	1.11	[1.06,1.16]	3.10E-05	0.03726	PFC
**DCP2**	1.1	[1.06,1.14]	4.23E-06	0.01056	PFC
**DZIP1**	1.12	[1.06,1.18]	5.97E-05	0.03726	PFC
**ETS2**	−1.09	[−1.13,-1.04]	5.36E-05	0.03726	PFC
**EVI2B**	−1.1	[−1.16,-1.05]	5.92E-05	0.03726	PFC
**FERMT2**	1.09	[1.05,1.14]	3.82E-05	0.03726	PFC
**FKBP5**	1.17	[1.08,1.26]	8.27E-05	0.04424	PFC
**LPIN1**	1.08	[1.04,1.12]	4.30E-05	0.03726	PFC
**TUFT1**	1.09	[1.05,1.13]	2.82E-06	0.01056	PFC
**UGT8**	−1.14	[−1.22,-1.07]	7.96E-05	0.04424	PFC
**WFS1**	1.1	[1.05,1.14]	1.26E-05	0.02359	PFC

## Discussion and conclusion

We report here the results of systematic review of gene expression studies in BP. BP is a complex disorder with a considerable genetic component that has been challenging to resolve. Gene expression studies may help to identify genes or sets of genes that are up or down regulated in the disorder and thereby provide clues about its genetic underpinnings. At least 30 studies using modern array-based technology to assay gene expression genome-wide have been published on BP. Most of these have studied expression in either blood or brain tissue samples. Although blood samples are easier to collect, brain samples provide more direct access to changes in the tissue most relevant to psychiatric disorders. We, therefore, conducted a quantitative mega-analysis of the most recent and robust of studies on the brain in BP in order to synthesize the findings and provide a comprehensive overview of what is currently known from these efforts.

The most significant findings were observed in the analysis of the PFC. This may reflect the central role the prefrontal cortex is thought to play in mood disorders, especially bipolar disorder [66]. However, it may also be due to the fact that the PFC was the focus of more studies than any other brain region. Although the analysis of any brain regions included more studies, these studies covered several different brain regions including the PFC, which may have introduced heterogeneity and diluted the findings. The analysis of the hippocampus only included two studies and was, therefore, relatively underpowered to detect differentially expressed genes.

In the PFC, there were 11 genes with a q-value < 0.05. Among these were two genes of great interest in mood disorders: *FKBP5* and *WFS1*. Mutations in *WFS1* are known to cause Wolfram syndrome, a disorder characterized by insulin deficiencies leading to high blood sugar levels and progressive vision loss, and which often co-occurs with psychiatric disturbances such as mood disorders. Several studies have directly implicated *WFS1* in the etio-pathogenesis of bipolar disorder [67]. *FKBP5*, on the other hand, encodes for FK506 binding protein 5, a co-chaperone of the glucocorticoid receptor heterocomplex, which mediates downstream effects of cortisol. The role of *FKBP5* and cortisol dynamics have been the focus of intense investigations in mood disorders [68,69] and response to antidepressant treatment [70,71]. Interestingly, *CRH* (corticotrophin releasing hormone) [72,73], another key gene underlying cortisol action, was identified as differentially expressed in the analysis of PFC. Several other notable candidate genes for mood disorders were implicated in the current analyses, including *DUSP6* (dual-specificity phosphatase 6) [74-76], *NPY* (neuropeptide Y), *NR4A2* (nuclear receptor subfamily 4, group A, member 2), *SST* (somatastatin), *GRIK2* (glutamate receptor ionotropic kainate 2 isoform precursor) [77-79], *S100B* (S100 calcium binding protein B) [80,81] and *CACNA1C* (calcium channel, voltage-dependent, L type, alpha 1C subunit). Perhaps of greatest interest among these is *CACNA1C*, which has emerged from recent genome-wide association studies as one of the leading candidate genes for bipolar disorder [82,83]. The MAPK gene, DUSP6, and neuropeptides, *NYP* and *SST*, are discussed further below.

Among the top findings from our pathway analyses were the up-regulation of metallothionein genes across any brain region and specifically in the PFC. This collection of genes was highlighted as significantly differentially expressed in several previous studies, including a weighted gene co-expression network analysis of BP and schizophrenia [84] and two previous meta-analyses of BP and psychosis using gene expression studies from SMRI [6,85]. The two meta-analyses included several studies that we excluded due to quality control measures, and we included one study on a unique set of brain samples that was not included in theirs. In addition, we used an entirely different approach for processing and analyzing the data. The fact that the results for the metallothionein proteins were sustained in multiple analyses lends support to the conclusion that the findings are real. Interestingly, studies with animal models have suggested the involvement of metallothioneins in neurocognitive function [86,87], and particularly in protecting the central nervous system against degeneration caused by various types of brain injury [88,89].

Also implicated in the pathway analysis were the mitogen-activated protein (MAP) kinase phosphotases. These are members of the dual specificity phosphatase (DUSP) family, which are known to negatively regulate members of the MAP kinase superfamily. MAP kinases have been shown to play a role in neuronal differentiation, neuronal survival, and long term neuroplasticity, and it has been suggested that lithium and valproate may exert therapeutic effects in BP by activating MAPK/ERK signaling cascades [90]. One of the key genes in the pathway identified by our current analysis was DUSP6, which was found to be significantly down-regulated in BP. DUSP6 is known to bind to and inactivate ERK1 and ERK2 [91], and previous studies have suggested a genetic association between DUSP6 and both schizophrenia and BP [74,75].

Another notable finding from our pathway analyses suggested there is a down-regulation of neuropeptides such as neuromedin U (*NMU*), neuropeptide Y (*NPY*), and somatostatin (*SST*) in BP. *SST*, in particular, was reported as significantly down-regulated in the other meta-analysis referenced earlier as well [84]. It was also implicated in a combined analysis of gene expression studies of the dorsolateral prefrontal cortex in schizophrenia [92], and in analysis of studies of the subgenual anterior cingulate cortex in major depression [93]. Neuropeptides are chemical messengers that are widely distributed throughout the peripheral and central nervous system, and they exert diverse effects in serving as hypothalamic releasing factors, neuromodulators, and/or neurotransmitters. There has been a great deal of interest in the role of neuropeptides such as neuropeptide Y and somatostatin in mood and anxiety disorders and as potential therapeutic targets [94].

It is noteworthy that the metallothioneins were not found to be significantly differentially expressed in the hippocampus. This may reflect differences in dysregulated gene expression patterns across different brain regions in BP, or it may be due to the fact that there were considerably fewer studies of the hippocampus resulting in relatively less power to detect meaningful differences. Clearly, further expression studies in this important brain region are needed.

The effort to synthesize findings from existing genome-wide expression studies of the brain in BP was complicated by several important challenges. First, there may be concerns about combining results across potentially heterogeneous studies. For example, studies of gene expression in the brain have used a variety of array platforms and examined different regions of the brain, which might contribute to the heterogeneity. In order to minimize such concerns, we included only the most recent and most comprehensive studies that all used a comparable array platform, and we obtained the raw data from each of the studies and analyzed this data using a standardized pipeline. In addition, we conducted separate mega-analyses for key regions of the brain.

Second, multiple studies were carried out using overlapping brain samples. Because of the challenges in collecting post-mortem brain tissue, there are limited such samples. Indeed, available samples have essentially come from 4 brain banks, and these have been studied multiple times by different research groups. Unfortunately, data from two of the existing brain banks were not available. We sought to use whatever data was available, and we used an analytic approach that appropriately handled the correlation induced within studies and within samples used across multiple studies.

Third, there may be many factors that confound the relationship between gene expression levels in post-mortem brain samples and disease status. Pre-mortem exposures and treatment histories, especially pharmacologic, may vary between cases and controls and drive differences in gene expression observed in brain samples. Likewise, post-mortem factors such as the agonal state, post-mortem interval between death and sample extraction, or sample pH may further degrade potential expression signals. Many of these factors may or may not be measured, and thus are difficult to correct [95]. We used an analytic approach that did not require all of the factors to be measured to account for this as best as possible. In particular, we used surrogate variable analysis which has been shown to be a powerful method for removing unwanted measured and unmeasured sources of heterogeneity [30]. However, it is possible this approach did not completely correct for all sources of heterogeneity, which may have confounded the findings.

Despite the challenges, our analyses provide an up-to-date summary of results from expression array data in BP. These analyses focused on the highest quality non-redundant data available and provides results by brain region so that similarities and differences can be sought that might be relevant to disease status. The results are available for closer inspection on-line at Metamoodics [http://metamoodics.igm.jhmi.edu/], a bioinformatics resource that we have created to gather results from genomic experiments in mood disorders. Investigators can look up any genes of interest and view the current results in their genomic context and in relation to leading findings from other genomic experiments in bipolar disorder.

## Abbreviations

BP: Bipolar disorder; MD: Major depression; SCZ: Schizophrenia; CTRL: Controls; PFC: Pre-frontal Cortex; FC: Fold change; CI: Confidence interval; RNA: Ribonucleic acid; GEO: Gene expression omnibus; ID: Identifiers; FLEO: Feature level extraction output; GEDM: Gene expression data matrix; fRMA: Frozen robust multi-array analysis, MAS5; RMA: Robust multi-array average; gcRMA: gc robust multi-array average; MBEI: Model based expression index; PLIER: Probe logarithmic intensity error; PANP: Presence absence calls with negative probesets; PM: Perfect match; MM: Mismatch; NSMPs: Negative strand matching probesets; EST: Expressed sequence tag; P: Present; A: Absent; M: Marginal; SVA: Surrogate variable analysis; FDR: False discovery rate; SMRI: Stanley Medical Research Institute/Stanley Foundation; SMRI (A): Array collection; SMRI (C): Neuropathology collection; HBTRC: Harvard brain tissue resource center (McLean Hospital, Belmont, Massachusetts); QSBB: Quebec suicide brain bank; cDNA: Complimentary deoxyribonucleic acid.

## Competing interests

The authors declare that they have no competing interests.

## Authors’ contributions

FS, MP and PPZ participated in the design of the study. FS performed the statistical analysis. FS, MP, FSG, JJ, JBP and PPZ conceived of the study, and participated in its design and coordination and helped to draft the manuscript. All authors read, contributed to and approved the final manuscript.

## Pre-publication history

The pre-publication history for this paper can be accessed here:

http://www.biomedcentral.com/1471-244X/13/213/prepub

## Supplementary Material

Additional file 1 **Results of qualitative review.** Details of 30 genome-wide gene expression array case–control studies of BP identified in our literature search. Click here for file

Additional file 2 **Results of 382 differentially expressed genes.** Details of 382 genes identified as differentially expressed with a regression beta estimate = ±0.1, equivalent to fold change (FC) > 1.07 (up-regulated) or FC < −1.07 (down-regulated) with p-value < 0.05 from the three mega-analyses: 191 in any brain region, 160 in the PFC, and 118 in the hippocampus; 80 of these were identified in more than one mega-analysis. Click here for file

Additional file 3 **Results of DAVID analysis.** Results of DAVID analysis showing an enrichment of common pathway annotations among the significant differentially expressed genes in the three mega-analyses i.e. 191 in any brain, 160 in the PFC and 118 in the hippocampus. Pathways and annotation categories included were the biological biochemical image database (BBID), BIOCARTA, KEGG_PATHWAY and Gene Ontology. Several gene categories were identified with a Bonferroni corrected p-value < 0.05 across the three mega-analyses. We provide two scripts for performing the following analyses on your own data:1. Gene expression data processing step. 2. Mega-analysis step. Scripts can be downloaded from: http://psychiatry.igm.jhmi.edu/geneexpression/. Click here for file
